# Medical Opinion and Movement

**Published:** 1909-07-31

**Authors:** 


### Medical Opinion and Movement.

An exhaustive inquiry into the Geographical Distribution of Diabetes Mellitus appears in the *Medical Chronicle*, from the pen of Dr. R. T. Williamson. While diabetes can and does occur in all climates and races, statistics and experience prove that its frequency varies very greatly in different parts of the world. The greater incidence of the disease upon Jews than upon European races has long been known; but it may not be generally appreciated that their death-rate is six to seven times that among Gentiles from this malady. Such at least is the case in Buda Pesth, where the statistics have been very carefully worked out; and the same holds good of Frankfurt also. The tendency to obesity in middle-aged Jews may possibly be correlated with the frequency of diabetes. In America negroes suffer decidedly less than white people, though the discrepancy is getting less as their habits and diet approximate to those of the latter. Hindus, Cinghalese, and Maltese suffer far more than European races, broadly speaking, whereas in Chinese on their own native diet, and in Eskimos, the malady is hardly ever seen. Thus a strictly carbo-hydrate diet cannot be regarded as necessarily disposing to diabetes; yet all the same, there are reasons for believing that many of those affected might not have developed glycosuria had excess of sugar in food or drink not been indulged in. It is suggested as worth inquiry whether a possible cause of the rapid increase of diabetes of late years is the widespread adoption of beet sugar in place of cane sugar.

---

The Correct Surgical Treatment of Abscesses in Hip Disease is still a matter concerning which individual surgeons differ widely. Dr. H. Schwatt in *International Clinics* lays down the following conclusions. Prompt evacuation of pus as soon as diagnosed does not rest upon a rational foundation, and should not be resorted to; but, at the same time, absolute non-interference is not to be made an invariable rule. The formation and extension of abscess can be averted in a large proportion of cases by effective treatment of the joint disease. To this end he recommends principally fixation of the joint, rest in bed with extension, tonic drugs, diet, and good hygiene. Operative interference when necessary is not to be regarded as a curative procedure for either the abscess or the underlying disease, and is usually followed by mixed infection. Interference with the wall of the abscess should be avoided on account of the danger of disseminating the tuberculous process, and therefore operation should consist simply of incision and evacuation of pus. Closure of the wound by suture may be attempted, but permanent primary union is not obtained as a rule, and in the majority of cases it is probable that drainage would be beneficial to the bone disease. Mixed infection is to be guarded against by scrupulous asepsis at operation and in the after treatment. The author adds a plea for a more conservative attitude towards this complication of hip disease than at present prevails.

An interesting article on Ruptures of the Œsophagus appears in a recent number of the *Beiträge zur Klin. Chirurgie* from the pen of Petren. The author recognises two classes of rupture—that which is the result of pathological conditions, such as cancer, ulceration, suppuration, aneurysm, etc., and that which follows trauma, without any pre-existing disease. It is with this traumatic variety that the author is chiefly concerned. He has found five cases recorded in which rupture occurred as the result of violent trauma to the whole body without there being any external wound; but as a rule it is met with in healthy individuals either as the result of violent vomiting or during a fit of suffocation. Rupture has, however, been found in the course of cerebral and abdominal affections. The site of injury is usually the lower end of the œsophagus, and it may extend into the cardiac end of the stomach. The tear is as a rule longitudinal, and occurs most frequently posteriorly or laterally, varying in length from one-fifth to two and a half inches. The edges of the wound are clean-cut, and usually no signs of previous disease are to be found. Symptoms vary in different cases, but in the majority the subjects are young or middle-aged and intemperate in matters of food and drink. During an attack of vomiting or suffocation following a heavy meal or an alcoholic debauch, the patient is seized with violent epigastric pain and a sense of internal tearing. Symptoms of collapse rapidly ensue, such as cold sweats, pallor, feeble pulse, and dyspnœa, and in half the cases there is vomiting and even hæmatemesis. The characteristic symptoms supervene later, when subcutaneous emphysema by extension from the cellular tissue of the mediastinum commences to appear, the asphyxial and agonising symptoms increasing, the heart beating feebly, and death ensuing in from seven to thirty hours.

---

Petren believes that Rupture of the Œsophagus is brought about by the sudden pushing back of the stomach-contents towards the œsophagus, partly by the powerful contractions of the abdominal muscles, which injure the organ, and partly by the congestion produced directly by the injury to the upper part of the abdomen. He is not of opinion that any antecedent disease is necessary to produce the rupture, though, of course, this latter would be facilitated if disease were present. In the particular case which drew his attention to the study of rupture of the œsophagus no such antecedent disease was present. A healthy young man, while working, accidentally received into his mouth air, under a pressure of seven atmospheres, which was emerging from a pipe. The lower end of his œsophagus was ruptured, mediastinitis set in, followed by left-sided pleurisy, progressive emphysema of the cellular tissues of the mediastinum and neck, and death at the end of 27 hours from asphyxia and cardiac failure.

THE Frequent Failure of the Urine to Decompose in cases of Pulmonary Tuberculosis is the subject of a research by Drs. Hale White and Janmohamed published in the *Quarterly Journal of Medicine*. Eight cases of undoubted pulmonary tuberculosis, in each of which tubercle bacilli were demonstrated in the sputum, were observed; and twenty-nine specimens were collected, each of the whole twenty-four hours' output. On estimating the acidity with decimormal soda it appeared that, in the main, the urine of those with pulmonary tuberculosis is much more highly acid than is that of sufferers from other diseases. Further, among the control patients two exhibited tuberculous peritonitis, and one Addison's disease, in each case without any demonstrable involvement of the lungs; and these three patients passed urine of no abnormal acidity. As all the patients with tuberculosis of the lungs were in-patients and severely ill, it was thought that possibly the failure of the urine to decompose and its high acidity were common to any severe infective disease. But the examination of four cases of pneumonia and one of septicæmia disposed of this: for all these urines decomposed rapidly, and their acidity, though more than normal owing to concentration, was much less than that of the urines of the tuberculous patients, which moreover were of low rather than high specific gravity. No constant relation was found between the degree of acidity and the length of time before decomposition of the urines. It was further established that the urine of two of the patients under observation was sterile; and then its bactericidal power was tested, and proved to be fairly considerable for the bacillus coli communis. Yeasts and moulds, on the other hand, flourish freely in such urine. No proof of the presence of any opsonins towards either the tubercle bacillus or the bacillus coli could be established. It should be mentioned that no drugs were given throughout the investigations.

AN attempt has been made by Dr. A. J. Hall to estimate as exactly as possible the Effects of Certain Drugs in Diabetes Mellitus. For this purpose he treated nine diabetic patients in hospital wards under conditions as nearly as possible identical, and in each case he varied the factors of their treatment—rest, diet, and drugs—but one at a time. He began in each case by allowing ordinary diet, with rest in bed and no drugs. When the body weight, and output of urine and sugar had settled down to fairly regular figures, a change was made to strict diet, and the results carefully tabulated; then some drug was exhibited, and after omitting it a period of time was always allowed to elapse before another was tried. Codeia was given to seven of the patients, beginning with $\frac{1}{4}$ grain three times a day, and increasing gradually up to doses of 4 grains. In some cases there was diminished secretion of urine, in others not; and similar disappointing results upon the output of sugar are recorded. In some cases large doses of codeia actually seemed to increase glycosuria, and in others there was no marked effect either way. No complaint of craving was made when the drug was discontinued. Opium gave slightly more satisfactory results, and in three cases this was noticeable when codeia had failed: but one patient got worse and died while taking opium. The largest quantity given was 12 grains per day. Secretin was then tried in doses rising to 9 drachms a day: no benefit accrued. The same is true of aspirin, given for twenty-seven days to one patient in doses of 1 drachm per day. After complete rest for a week or two it was observed that some of the patients tolerated carbohydrate diet much better than when allowed up, the quantity both of urine and sugar diminishing; the body weight in these cases fluctuated a good deal.

THE Use of Scopolamine-Morphine Narcosis in Labour, first advocated by Kesnig, has been extensively tested and reported upon since by many obstetricians. Sir Halliday Croom, in the *Journal of Obstetrics and Gynæcology of the British Empire*, gives his experiences, which are entirely favourable. Beginning with a combination of 1-400th gr. of scopolamine and $\frac{1}{6}$ gr. of morphine, he found the analgesic results not quite satisfactory; and thirst was much complained of as an after-effect. After trying other proportions, he finally adopted $\frac{1}{100}$ and $\frac{1}{6}$ gr. as the best respective quantities, and of this dosage he reports that it markedly diminishes, and in some cases entirely abolishes, the pain of the uterine contractions. The patients sleep soundly between the pains, and in most cases for one or two hours after the completion of labour. Out of 62 cases, some narcosis was evident in the infant when born in 30 per cent., but only in two instances was there any difficulty in reviving the children, not one of whom was lost. Curiously, when complete anæsthesia has not been produced, there is yet very frequently loss of all remembrance afterwards of the labour pains. The drugs are given in the second stage of labour, and in most cases one dose suffices: a second dose, however, was given in about 40 per cent. of the cases without ill effect. Sir Halliday advises that for forceps, version, repair of perineum, and so forth a little chloroform should be given in addition; and he adds that for most multiparæ the latter suffices without scopolamine-morphine, which he especially recommends for primiparæ, and particularly for those of highly nervous temperament.

ORGANIC Iron is often tolerated in much larger quantities than inorganic iron compounds are, and this fact has led to the investigation of large numbers of different vegetables and plants from the point of view of the percentage of iron contained in their dry residue. Tabouriech and Saget have recently carried out extensive analyses upon the subject, and they have discovered that the dried root of the *Rumex obtusifolius*—the broad-leaved dock which must be familiar to most people in the country—contains no less than 0.447 per cent. of iron. This is a percentage which far exceeds any yet found in other plants. The iron is, of course, in organic combination, and the results obtained by exhibiting the powdered root of the *Rumex* by the mouth are excellent.

Writer's Cramp is said to be one of the many ailments of a local nature that may yield to Bier's treatment of hyperæmic congestion by the elastic bandage. Three years ago Dr. P. Hartenberg, of Paris, reported a case he had successfully treated in this way. The patient, a man aged 37, had suffered from writer's cramp for 15 years. Every means of treatment had been tried without success, and the condition was regarded as incurable. Without changing the habits or work of the patient, an indiarubber tube was tied around the arm above the biceps, and left in position for 20 minutes night and morning. At the end of a fortnight there was considerable improvement in the condition, and two months later the patient was almost cured. Recently the same treatment has been adopted by Dr. Bucciante, an Italian military surgeon in a similar case that had resisted the usual methods of treatment. An elastic bandage was placed around the middle of the arm for half an hour night and morning. Rapid improvement followed the treatment and at the end of three weeks the condition was said to be completely cured.

---

Dr. Balzer of the Saint Louis Hospital, and Dr. Mouneyrat, Professor of Medicine at Lyons, have presented to the *Societé Médicale des Hôpitaux*, an important report upon a new organic compound of arsenic, called Hectine, which appears to have remarkable therapeutic properties in cases of syphilis. It has a complex constitution, and is described chemically as the sodium salt of benzo-sulphone-para-aminophenylarsenic acid. It is characterised by its great solubility in water, and the facility with which it combines with mercurial and iodine compounds. It is said to be much less toxic than atoxyl and its derivatives. Its low degree of toxicity has been verified in animals—mouse, rabbit, and dog, and in man, and it does not produce ocular troubles. Its elimination is very rapid during the first two or three days of administration, and then gradually becomes slower, so that it is advised to administer the drug by injections, or by the mouth every day, or every other day for several days, with intervals of rest of about 10 days. For adults as much as 10 to 20 centigrammes may be given daily. The injections are said to cause little or no pain, and are not followed by inflammatory or other troubles. The authors have used both hectine and hectargyre (the mercurial preparation) in all forms of secondary and tertiary syphilis, and report most enthusiastically of the rapid manner in which the disease yields to the action of these preparations.

---

Some interesting observations were carried out by Dr. D. De Sandro, at the Naples Hospital, on the urine of 320 patients admitted for injuries sustained during the Messina earthquake. Among them there were 40 cases of fracture of long bones, nine cases of injury to the spine, three abdominal injuries, and one probable fracture of the base of the skull. The rest consisted of injuries to the soft parts in different regions of the body. The urinary analysis was limited to estimations of uric acid, lactic acid, glucose, and the urinary toxicity. One hundred and twenty-nine cases, or 40 per cent., showed a high specific gravity with marked excess of uric acid. This excess continued from admission (third to fifth day after the accident) to the 10th or 15th day, was independent of any uric acid diathesis, and was not accompanied by diminution in volume of urine excreted. It was not associated with any particular injury, but appeared to be closely connected with the extent of the injuries, and their multiplicity, and especially with the time during which the patients were confined among the *débris*. In regard to this last condition the author suggests that the excess of uric acid may be connected with the excessive muscular exertion in the efforts of the patients to disengage themselves from the *débris*, or with the respiratory distress consequent on more less complete immobilisation of the thorax. Lactic acid was found present in nine cases, or 2.8 per cent. In all these cases there was also excess of uric acid, so that of the 129 cases with uric acid excess 7 per cent. excreted also lactic acid. All these nine cases had remained some time under the *débris*, and three of them sustained considerable cranial injuries. Only six patients showed the presence of glucose in the urine, and they all belonged to the category of 73 cases of head injuries. It was present in the case of probable fracture of the base.

---

The *bruit d'airain* produced by the click of one coin upon another is a well-established diagnostic sign of pneumothorax, but the *signe du sou* described by Pitres in 1881 as a clinical sign in cases of pleuritic effusion has been almost entirely ignored. This *signe du sou* forms the subject of an interesting contribution to *Le Progrès Medicale* by Professor Henri Verger of Bordeaux, in which the author explains the value of this sign in examinations of the chest. This *signe du sou* is obtained in the same way as the *bruit d'airain*. A coin is placed on the chest and knocked upon with a second coin, while the physician applies the stethoscope at some opposite point. If the sound is transmitted through a continuous homogeneous layer, either solid or liquid, a clear metallic sound is heard. On the other hand, if the sound is transmitted through spongy tissue or through successive layers of different composition, the sound is heavy and muffled. By this sign the author claims that fluid at the base of a lung can frequently be diagnosed, in cases, for instance of cardiac failure, when it is otherwise often impossible to distinguish from pulmonary congestion, except by the exploring needle. He considers this *signe du sou* the most constant and most sure of all the known signs of pleuritic effusion, and by it the variations in the upper limit of the fluid during the course of the disease can be most easily determined. On the other hand, in cases when pulmonary adhesions prevent the usual elevation of the lung with the increase in the effusion, the sign is naturally more equivocal, but even in such cases it may prove more definite than the usually accepted diagnostic signs. In cases of putrid pleurisy, where an early diagnosis is so essential, the routine use of this *signe de sou* at once gives the *bruit d'airain* due to gas in the pleuritic cavity, which may otherwise not be sought for.

